# Impact of the COVID-19 Pandemic on Loneliness Among Older Adults

**DOI:** 10.3389/fsoc.2020.590935

**Published:** 2020-10-30

**Authors:** Alexander Seifert, Benedikt Hassler

**Affiliations:** School of Social Work, Institute for Integration and Participation, University of Applied Sciences and Arts Northwestern Switzerland, Olten, Switzerland

**Keywords:** social isolation, SARS-CoV-2, social contact, Switzerland, older adult, COVID-19, corona

## Abstract

The COVID-19 pandemic has created a pattern of everyday physical distancing worldwide, particularly for adults aged 65+. Such distancing can evoke subjective feelings of loneliness among older adults, but how this pandemic has influenced that loneliness is not yet known. This study, therefore, explored the association between subjective loneliness and different time phases of the COVID-19 pandemic to explain the pandemic's impact on loneliness among older adults. The analysis employed a sample of 1,990 community-dwelling older adults aged 65–95 (mean age = 72.74 years; 43% female) in Switzerland. Data collection occurred both before and after Switzerland's first confirmed COVID-19 case. Regression models allowed the researchers to determine the binary and multivariate effects of different pandemic time phases on loneliness. The descriptive analysis revealed that loneliness increased after the Swiss government recommended physical distancing and slightly decreased after the Federal Council decided to ease these measures. According to the multivariate analysis, women, lower-income individuals, individuals living alone, individuals with no children, individuals unsatisfied with their contact with neighbors, and individuals interviewed after the physical distancing recommendations were more likely to report greater loneliness. The results suggest the pandemic has affected older adults' subjective evaluations of their subjective loneliness, and these findings help illustrate the pandemic's outcomes.

## Introduction

The current coronavirus disease 2019 (COVID-19) and the governmental recommendations stemming from it have created a pattern of physical distancing worldwide, particularly for adults aged 65+. Millions of people either have been or remain quarantined in their homes as countries have implemented physical distancing measures to contain COVID-19 infections. This social isolation can lead to feelings of loneliness, which, if prolonged, can be detrimental to mental health and well-being (Banerjee and Rai, 2020).

Although previous research has shown that subjective loneliness can be intensified in older adults by negative, stressful situations (Hensley et al., 2012), less is known about the COVID-19 pandemic's influence on loneliness in this population (Vahia et al., 2020). This research, therefore, investigated differences in loneliness before and during the pandemic to explain COVID-19's effects on subjective loneliness among adults aged 65+ in Switzerland.

### Theoretical Assumptions

Loneliness is a complex psychosocial concept (Dykstra, 2009). This study defined loneliness as “the unpleasant experience that occurs when a person's network of social relations is deficient in some important way, either quantitatively or qualitatively” (Perlman and Peplau, 1981, p. 31). Loneliness can, therefore, be considered the subjective feeling of lacking social contact. Previous research has shown that socially isolated persons are at a greater risk of loneliness (de Jong Gierveld et al., 2006). However, socially isolated people are not necessarily lonely, and lonely people are not necessarily socially isolated (Hawkley and Cacioppo, 2010). Where people rest on the subjective loneliness continuum depends on their expectations and current situations (Dykstra, 2009).

Age is not a valid predictor of loneliness; nevertheless, meaningful social contacts are important for healthy aging (Holmén and Furukawa, 2002). COVID-19 has confronted older adults, first, with social isolation and, second, with the stress of not seeing family or friends and of finding themselves in the “at risk group.” Stressful reminders of “being in need” may produce negative self-perceptions, resulting in loneliness (Hwang et al., 2020).

### Research Aim

This research investigated the association between subjective loneliness and different time phases of the pandemic to explain COVID-19's impact on subjective loneliness among adults aged 65+. The authors expected that individuals interviewed after Switzerland's first governmental recommendations for maintaining physical distancing would report greater subjective loneliness.

## Materials and Methods

### Participants

This study was based on data from a representative survey (Swiss Survey 65+) of 1,990 adults aged 65+ living in Switzerland. The survey initially focused on older adults' resources for maintaining autonomy in their own households; therefore, it was not constructed as a pandemic-related survey. Nevertheless, data collection occurred both before and after the first confirmed COVID-19 case in Switzerland (February 25, 2020), the first confirmed COVID-19-related death in Switzerland (March 5, 2020), and the Swiss Federal Council's decision (March 16, 2020) to introduce “extraordinary situation” measures for public protection.

From January to May 2020, 1,900 people aged 65+ were interviewed using a computer-assisted telephone interview (CATI) approach supplemented by paper-and-pencil surveys. The mean age of the sample was 72.74 years (SD: 5.18; age range: 65–95), and 42.8% of respondents were female. Based on media releases from the Swiss Federal Office of Public Health (FOPH) regarding the government's COVID-19 response, the sample was divided into four subgroups according to interview date.

- Phase 1 (Jan 27–Mar 6): Start of survey to Federal Council “call for special protection of older adults” (FOPH, 2020a), (*n* = 391).
- Phase 2 (Mar 7–Mar 16): Up to Federal Council declaring an “extraordinary situation” (FOPH, 2020b), (*n* = 582).
- Phase 3 (Mar 17–Apr 8): Up to Federal Council deciding to gradually ease the shutdown (FOPH, 2020c), (*n* = 757).
- Phase 4 (Apr 9–May 5): Up to the end of data collection, (*n* = 260).

Table 1 describes the sample and subgroups.

**Table 1:** Descriptive characteristics of the sample and subgroups.

**Parameter**	**Scale**	**Study sample (*N* = 1,990)**	**Subgroup 1 [Jan 27 to Mar 6] (*n* = 391)**	**Subgroup 2 [Mar 7 to Mar 16] (*n* = 582)**	**Subgroup 3 [Mar 17 to Apr 8] (*n* = 757)**	**Subgroup 4 [Apr 9 to May 5] (*n* = 260)**
		**% or mean**	**% or mean**	**% or mean**	**% or mean**	**% or mean**
Gender	Female	42.8	56.0	33.5	37.1	60.4
	Male	57.2	44.0	66.5	62.9	39.6
Age	Mean^a^	72.74	74.51	72.10	72.24	72.95
Living alone	Yes	26.5	30.7	23.8	27.5	23.3
	No	73.5	69.3	76.2	72.5	76.7
Education	Mean^b^	2.94	3.18	2.93	2.77	3.04
Income	Mean^c^	5.33	5.11	5.56	5.36	5.14
Children	Yes	85.6	88.0	85.6	85.1	83.8
	No	14.4	12.0	14.4	14.9	16.2
Living area	Non-rural	76.7	69.3	78.4	80.2	74.2
	Rural	23.3	30.7	21.6	19.8	25.8
Satisfaction about contact with neighbors	Mean^d^	7.90	7.98	7.91	7.74	8.22
Loneliness	Mean^e^	1.79	1.69	1.78	1.84	1.79

a *Age range: 65–95*;

b *Education scale (1 = preprimary education, 5 = second state of tertiary education)*;

c *Income scale (1 = up to 1,200 CHF, 9 = over 15,000 CHF)*;

d *Satisfaction with contact with neighbors (0 = completely dissatisfied, 10 = completely satisfied)*;

e *Loneliness scale (1 = low, 5 = high)*.

### Measures

Subjective loneliness was assessed via a shortened, six-item version of the (de Jong Gierveld and van Tilburg, 1999) Loneliness Scale: *There are plenty of people I can lean on when I have problems; I often feel rejected; There are many people I can trust completely; I miss the pleasure of the company of others; There are enough people I feel close to; I miss having a really close friend*. Participants answered the items on a five-point scale (1 = does not apply at all, 5 = fully applies). The six items loaded on one factor, with factor loadings from 0.63 to 0.74. Cronbach's alpha for the scale was 0.783. The mean (M: 1.78, SD: 0.724) of all items was calculated, with higher scores reflecting greater loneliness. Time-related subgroups were selected via FOPH media releases, as described above.

Covariates, evaluated as important loneliness predictors in previous research (Vozikaki et al., 2018), included chronological age in years; sex (0 = male, 1 = female); educational level (1 = preprimary education, 5 = second state of tertiary education); monthly household income (1 = up to 1,200 CHF [Swiss francs], 9=over 15,000 CHF); living alone (0 = no, 1 = yes); having children (0 = no, 1 = yes); living in a rural area (0 = no, 1 = yes); and overall subjective satisfaction about contact with neighbors [one item(“How satisfied are you about your contact with your neighbors?”) measured on an 11-point scale (0 = completely dissatisfied, 10 = completely satisfied)].

### Analytical Strategy

First, the Loneliness Scale mean values, divided into the four time-related subgroups, were graphically presented to show value changes. Second, single regression models were calculated to determine the binary effects of all independent variables on loneliness. Third, a multiple hierarchical linear regression model was employed to analyze the predictors of loneliness. Missing data were excluded. All analyses were conducted using SPSS 26 software.

## Results

**Figure 1:**
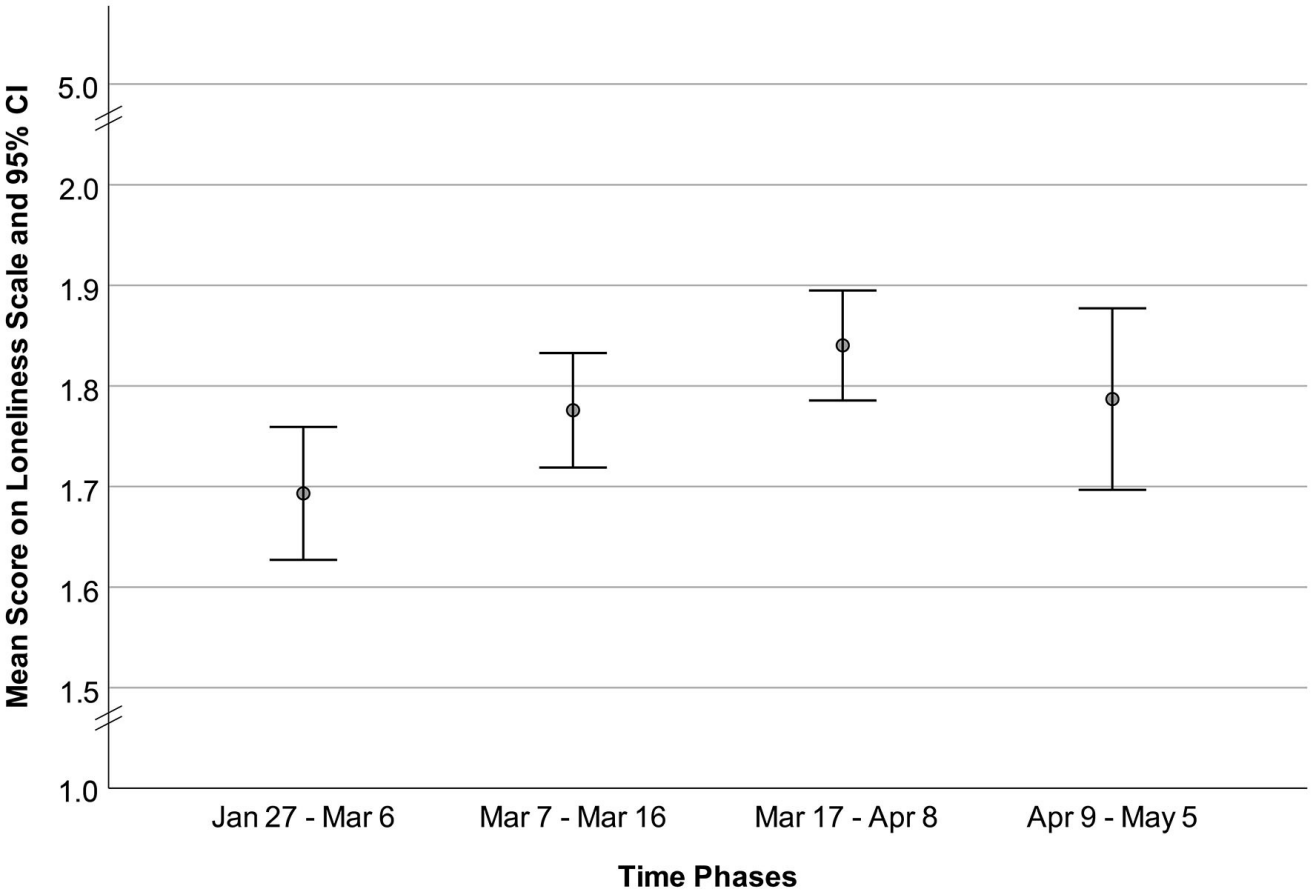
Differences in loneliness between the four time groups.

Table 1 shows the descriptive statistics for the sample and the four subgroups. Figure 1 presents the Loneliness Scale means for each time-related sub-group. Loneliness increased from the first to third subgroups and decreased from the third to fourth subgroups.

Table 2 shows the bivariate relationships between the four subgroups and the covariates with the independent variable “loneliness.” In the single gross models, loneliness was statistically significantly associated with the differences between the first subgroup and the third subgroup, revealing that the increase in loneliness was significant for comparing the first subgroup to the third subgroup. All covariates, except age, gender, and rural area, were statistically significantly associated with loneliness.

**Table 2:** Linear Regression analyses with loneliness as dependent variable.

**Parameter**	**Scale**	**Single gross models**	**Model A: standard demographics**	**Model B: living situation**	**Model C: full model**
		**Beta**	**Beta**	**Beta**	**Beta**
Age	65–95	0.026	0.019	−0.023	−0.008
Gender	Female (ref. male)	−0.015	0.003	0.050^*^	0.059^*^
Education	1–5	−0.086^***^	−0.005	−0.047	−0.036
Income	1–9	−0.180^***^	−0.183^***^	−0.064^*^	−0.073^**^
Living alone	Yes (ref. no)	0.217^***^		0.198^***^	0.198^***^
Children	Yes (ref. no)	−0.112^***^		−0.060^**^	−0.059^*^
Rural area	Yes (ref. no)	−0.029		−0.006	0.002
Satisfaction about contact with neighbors	0–10	−0.367^***^		−0.371^***^	−0.369^***^
Subgroup 2 [Mar 7–Mar 16]	(ref. subgroup 1 [Jan 27–Mar 6])	0.052			0.089^**^
Subgroup 3 [Mar 17–Apr 8]	(ref. subgroup 1 [Jan 27–Mar 6])	0.099^**^			0.095^**^
Subgroup 4 [Apr 9–May 5]	(ref. subgroup 1 [Jan 27–Mar 6])	0.044			0.064^*^
Model fit			F (4, 1638) = 14.811; *p* < 0.001; *R^2^* = 0.035	F (8, 1609) = 52.593; *p* < 0.001; *R^2^* = 0.208	F (11, 1609) = 39.602; *p* < 0.001; *R^2^* = 0.214

*Dependent variable: Loneliness scale (scale 1–5)*;

* *p < 0.05*;

** *p < 0.01*;

*** *p < 0.001*.

Table 2 also shows the hierarchical linear regression analyses for the multivariate predictors of loneliness. In model (A), only income was a statistically significant loneliness predictor. In model (B), gender, income, living alone, having children, and being satisfied about contact with neighbors were statistically significant loneliness predictors. In the full model (C), subgroups two through four, compared to the first subgroup and the same covariates as in (B), were statistically significant loneliness predictors. Females, individuals with lower incomes, individuals living alone, individuals with no children, individuals who were dissatisfied about their contacts with neighbors, and individuals who were interviewed after March 6, 2020, were more likely to report greater loneliness.

Supplementary analysis addressed potential differential findings with other subgroups as references in the linear regression analysis. Findings from those additional analyses confirmed the previous results; only the difference between the first time phase and time phases two through four significantly predicted loneliness (Table 3).

**Table 3:** Linear Regression Analyses with Loneliness in Comparison with Different Subgroups as Reference.

**Parameter**	**Model A: ref. subgroup 1**	**Model B: ref. subgroup 2**	**Model C: ref. subgroup 3**	**Model D: ref. subgroup 4**
	**Beta**	**Beta**	**Beta**	**Beta**
Subgroup 1 [Jan 27–Mar 6]	–	−0.082^**^	−0.081^**^	−0.079^**^
Subgroup 2 [Mar 7–Mar 16]	0.089^**^	–	0.001	0.004
Subgroup 3 [Mar 17–Apr 8]	0.095^**^	−0.001	–	0.002
Subgroup 4 [Apr 9–May 5]	0.064^*^	−0.003	−0.002	–
Model fit	F (11,1609) = 39.602; *p* < 0.001; *R^2^* = 0.214	F (11,1609) = 39.602; *p* < 0.001; *R^2^* = 0.214	F (11,1609) = 39.602; *p* < 0.001; *R^2^* = 0.214	F (11,1609) = 39.602; *p* < 0.001; *R^2^* = 0.214

*Dependent variable: Loneliness scale (scale 1–5); Controlled for: Age, gender, education, income, living alone, children, rural area, and satisfaction about contact with neighbors*;

* *p < 0.05*;

** *p < 0.01*;

*** *p < 0.001*.

## Discussion

This study explored the impact of different COVID-19-related time phases on subjective loneliness among adults aged 65+. As the authors hypothesized, loneliness was associated with the time periods in which the interviews took place. Individuals interviewed before the Federal Council called for the special protection of older adults reported lower loneliness than those interviewed later. Thus, the results suggest that the pandemic—more specifically, the Federal Council's call for the special protection of older adults through physical distancing—affected older adults' subjective evaluations of their loneliness.

Subjective loneliness increased between the first and second and between the second and third subgroups, but it slightly decreased, as a possible “normalization” of loneliness, after the Federal Council called for easing the official COVID-related restrictions. Other recent studies have also found these up-and-down movements, indicating that loneliness increased during the first weeks of the COVID-19 lockdown and decreased thereafter (Buecker et al., 2020; Höglinger et al., 2020). However, the present data collection ended on May 5, 2020, and, therefore, further research is needed to evaluate future developments.

Nevertheless, from the available data, it may be assumed that recommendations for older people to maintain physical distancing directly or indirectly affected their loneliness—probably by (a) limiting social contact opportunities; (b) making older individuals reflect on their social/support networks, potentially evaluating them as frail; (c) labeling older adults as “at risk,” possibly causing them to be shunned; and (d) making older individuals feel lonely because society considered them old and frail and, therefore, lonely.

In addition to this time-related effect, loneliness factors known from previous research—being female, having low income, living alone, having no children, and having no good contact with neighbors—were also found. COVID-19 has affected subjective loneliness, but this does not eliminate existing inequalities. Current research states that, during a pandemic, woman, and people with low incomes are likely to be loneliest (Bu et al., 2020). Therefore, existing inequalities among older adults should also not be neglected during the pandemic. Though the media often considers older adults as a homogeneous “at risk group,” this study found no age-related association with loneliness. Older adults should not be viewed as a homogenous “vulnerable” group, and undifferentiated, blanket measures that disproportionately exclude older adults are often based on grossly simplified age stereotypes, which can reproduce age discrimination (Ayalon et al., 2020; Losada-Baltar et al., 2020).

Furthermore, these results confirmed that not all older adults reported loneliness; those who did should be asked what could help them overcome those feelings. This calls for an individual—instead of a sweeping, group—view of loneliness, and gerontological social work responses must be tailored to individual needs (Berg-Weger and Morley, 2020).

Despite this study's strengths, several limitations must be noted. First, this research focused on Switzerland, so the findings have limited generalizability. Second, the existing data provided only a cross-sectional view. Third, because of the study variables' limited width, the authors could not control for other important background factors, such as measurements of quantity/quality and valuations of social contacts, personality, or attitudes toward COVID-19 governmental restrictions. Furthermore, those additional variables could help to set the new COVID-19-contextual findings in relation to the existing research literature about subjective loneliness among older adults. Clearly, further studies with longitudinal designs and wider variable ranges are required to examine this topic in more detail.

## Data Availability Statement

The raw data supporting the conclusions of this article will be made available by the authors, without undue reservation.

## Ethics Statement

Ethical review and approval was not required for the study on human participants in accordance with the local legislation and institutional requirements. Written informed consent for participation was not required for this study in accordance with the national legislation and the institutional requirements.

## Author Contributions

All authors listed have made a substantial, direct and intellectual contribution to the work, and approved it for publication.

## Conflict of Interest

The authors declare that the research was conducted in the absence of any commercial or financial relationships that could be construed as a potential conflict of interest.
